# Recent Developments in the Field of Endoscopic Ultrasound for Diagnosis, Staging, and Treatment of Pancreatic Lesions

**DOI:** 10.3390/cancers15092547

**Published:** 2023-04-29

**Authors:** Marie Poiraud, Paraskevas Gkolfakis, Marianna Arvanitakis

**Affiliations:** Department of Gastroenterology, CUB Erasme Hospital, Université Libre de Bruxelles, 1070 Brussels, Belgium

**Keywords:** endoscopic ultrasound, pancreatic cancer, artificial intelligence, tissue acquisition

## Abstract

**Simple Summary:**

The management of pancreatic lesions and especially of pancreatic cancer remains a diagnostic and therapeutic challenge given their poor overall prognoses. This review aims to summarize the available evidence on the role of endoscopic ultrasound regarding diagnosis, staging, and treatment of pancreatic lesions. Moreover, it highlights potential future research opportunities that could lead to improved patient outcomes.

**Abstract:**

Endoscopic ultrasound (EUS) plays a crucial role in the diagnosis of both solid and cystic pancreatic lesions and in the staging of patients with pancreatic cancer through its use for tissue and fluid sampling. Additionally, in cases of precancerous lesions, EUS-guided therapy can also be provided. This review aims to describe the most recent developments regarding the role of EUS in the diagnosis and staging of pancreatic lesions. Moreover, complementary EUS imaging modalities, the role of artificial intelligence, new devices, and modalities for tissue acquisition, and techniques for EUS-guided treatment are discussed.

## 1. Introduction

The field of digestive oncology and, in particular, the management of pancreatic tumors has made significant progress with the development of novel technical advances for improving diagnostic accuracy and staging. During the last few decades, endoscopic ultrasound (EUS) has evolved from a diagnostic tool to a therapeutic tool with the application of new devices and techniques [1]. Moreover, progress in the field of artificial intelligence (AI) has provided significant breakthroughs that have facilitated a more systematic approach in diagnostic EUS, enhancing the quality of EUS examinations and assisting in overall decision making [2].

Pancreatic ductal adenocarcinoma (PDAC) is associated with poor outcomes when diagnosed at the locally advanced or metastatic stage [3]. EUS plays a major role in the diagnostic work-up of pancreatic masses, particularly for lesions smaller than 20 mm [4], since early diagnosis may identify surgical candidates that can be offered the only curative strategy available at this time [5]. Although cystic pancreatic lesions are most often benign entities which do not require surgery, precise diagnosis of cyst type, with the help of EUS, and establishment of the potential for malignant transformation are crucial for determining management [6]. Finally, EUS-guided local treatment can be proposed in some clinical settings, such as preneoplastic lesions or other types of pancreatic neuroendocrine tumors (pNETs).

**Figure 1:**
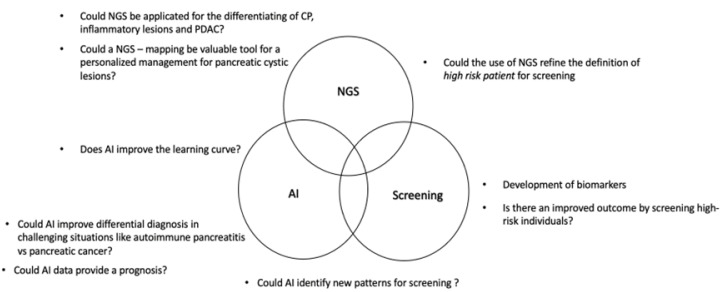
Fields for research perspectives.

The aim of this review is to present the most recent data regarding developments in the field of EUS that could offer complementary benefits in the management of pancreatic lesions. Figure 1 presents some of the future perspectives regarding the role of EUS for the management of pancreatic cancer.

## 2. Developments in the Diagnostic Role of EUS

### 2.1. Contrast-Enhanced EUS

Contrast-enhanced EUS (CE-EUS) is an add-on technique that uses a contrast agent, which creates microbubbles in the target tissue area once injected intravenously, in order to assess local micro-vascularization [7]. During its application, two main parameters are evaluated, enhancement and contrast distribution, that allow for a more meticulous evaluation of a solid lesion or cystic lesion with a solid component. CE-EUS can offer diagnostic arguments to differentiate chronic pancreatitis from PDAC with high sensitivity, specificity, positive predictive value, and negative predictive value, and is therefore retained as a reliable tool in discriminating PDAC from CP [8]. A recent retrospective study from Kataoka et al. [9] demonstrated the usefulness of CE-EUS in the differential diagnosis of solid pseudopapillary neoplasms and non-functional pNETs. Indeed, considering that about 30% of pNETs do not have typical identifying features [10], the differential diagnosis with solid pseudopapillary neoplasms is not always clear. In this study, the most accurate CE-EUS finding was the presence of the alveolus nest sign, which was significantly more frequent in solid pseudopapillary neoplasms compared to pNETs [10].

CE-EUS is also helpful in identifying worrisome features when assessing cystic lesions. For example, main pancreatic duct dilatation and the presence of enhanced mural nodules in cystic lesions represent a significant risk factor of malignancy in the setting of intraductal papillary mucinous neoplasm (IPMN) [11,12]. In a recently published meta-analysis, Lisotti et al. [7] showed that the sensitivity and specificity of CE-EUS for the diagnosis of mural nodules harboring high-grade dysplasia or invasive carcinoma were 88.2% and 79.1%, respectively. These numbers can further increase when a dedicated contrast-enhanced harmonic mode EUS (CEH-EUS) is used instead of Doppler mode (pooled sensitivity: 97%, pooled specificity: 90.4%), leading to an increased diagnostic yield [7].

In a recent prospective multicentric study, Omoto et al. [13] evaluated the role of tissue-harmonic EUS and CE-EUS for the diagnosis of PDAC, taking into consideration irregular periphery pattern and late phase hypo-enhancement. The authors demonstrated higher reproducibility for the diagnosis of PDAC using CE-EUS, with a diagnostic accuracy of 85.8% compared to 78.9% for tissue-harmonic EUS (*p* < 0.001).

Furthermore, Iwasa et al. [14] suggested using a combination of a qualitative analysis, using CEH-EUS, which provides information about the target tumor microvasculature, and quantitative analysis using the time-intensity curve. The analysis showed that the time-to-peak of malignant lesions was significantly shorter than that for benign lesions (*p* = 0.0009). Combining both analyses demonstrated a high diagnostic capacity in addition to EUS-fine needle aspiration (EUS-FNA) for diagnostic workup in solid pancreatic lesions.

In addition to increasing diagnostic capacity, CEH-EUS can also predict prognosis and aggressiveness of solid pancreatic lesions. According to Ishikawa et al. [15], hypo-enhancement on CEH-EUS is an indicator of aggressive pNETs, with sensitivity, specificity, positive predictive value, negative predictive value, and accuracy of 94.7%, 100%, 100%, 96.6%, and 97.9%, respectively. This initial data were confirmed by Constantin et al. [16] in a pilot study including 30 patients that aimed to predict prognosis of PDAC and pNETs using CEH-EUS. The authors reported that lower peak enhancement and lower wash-in area under the curve, assessed with CEH-EUS, were significantly associated with worse survival outcome in patients with PDAC [16]. Whether these techniques can be used as a surrogate for the risk of aggressiveness and support evidence-based clinical decision making remains an open question.

### 2.2. Elastography

Elastography is an additional assessment tool used in EUS procedures to evaluate tissue stiffness. Although initially promising, elastography remains a non-specific evaluation method that has not shown convincing results in terms of correlation between stiffness and the presence of neoplastic tissue; thus, it could be used only in complementary manner during the patient’s work up [17]. Nevertheless, the recent study from Ohno et al. [18] was conducted to evaluate EUS-elastography-guided fine needle biopsy for the diagnosis of solid pancreatic lesions. The results showed that elastography may reflect tissue composition in pancreatic solid lesions. However, it did not affect either the quality nor the quantity of the obtained tissue. Finally, another recent study by Gheorghiu et al. [19] reported no difference in the diagnostic yield of elastography-guided EUS fine-needle aspiration compared to B-mode EUS-FNA.

### 2.3. Needle-Based Confocal Laser Endomicroscopy and Microforceps Biopsies

Additional diagnostic techniques, such as needle-based confocal laser endomicroscopy (nCLE) and through-the-needle microforceps biopsy, have been recently evaluated, particularly with regard to assessment of cystic pancreatic lesions.

In a recent retrospective study, Robles-Medranda et al. [20] compared EUS and associated techniques such as EUS-FNA, CE-EUS, EUS-guided fiberoptic probe cystoscopy, microforceps biopsy, and nCLE for the detection of potentially malignant pancreatic cystic lesions. Malignancy detection was significantly more accurate with nCLE [OR 8.441] and microforceps biopsy [OR 3.425] than cystoscopy [OR 0.622]. When the three techniques were simultaneously performed (EUS with nCLE and microforceps biopsy), the diagnostic accuracy analysis showed that the sensitivity, specificity, positive predictive value, and negative predictive value were 100%, 89%, 78%, and 100%, respectively. Concerning nCLE, these results were consistent with those from a previous study from Napoléon et al. [21], which reported excellent diagnostic performance for confirming diagnosis of serous cystadenoma, which surpassed that of cystic fluid carcinoembryonic antigen (CEA) measurement for the diagnosis of large, single, noncommunicating cystic pancreatic lesions. Moreover, in a study conducted by Krishna et al. [22], EUS-guided nCLE criteria for the classification of IPMNs were assessed for the differentiation of high-grade dysplasia/adenocarcinoma and low/intermediate-grade dysplasia. The authors demonstrated that papillary epithelial width and darkness were able to accurately predict high-grade dysplasia. Despite these promising results, further studies are required to define the role of these emerging novel diagnostic tools in the clinical management of pancreatic cysts.

### 2.4. Cystic Fluid Analysis and Genetic Analysis

Regarding cystic fluid evaluation, it was recently shown that intracystic glucose levels are superior to CEA levels for differentiating mucinous neoplastic cysts from other cysts [23]. The cut-off for glucose levels was ≤25 mg/dL, which demonstrated a sensitivity of 88.1% and a specificity of 91.2%. It was also shown that the combination of glucose level and CEA did not perform better than glucose alone. Furthermore, the analysis of DNA mutations isolated from cystic fluid is a subject of interest and, in particular, KRAS and GNAS mutations have demonstrated good accuracy for the diagnosis of IPMNs and mucinous neoplastic cysts, as shown in a recent meta-analysis by MacCarty et al. [24]. The pooled sensitivity, specificity, and diagnostic accuracy of combining both KRAS and GNAS mutations for the diagnosis of IPMNs was 94%, 91%, and 97% respectively, with each significantly higher when compared with CEA alone [24]. Moreover, the combination of KRAS and GNAS had a significantly higher diagnostic accuracy than each mutation alone in patients with IPMNs and mucinous neoplastic cysts [24].

A recent multi-center prospective study from Paniccia et al. [25] demonstrated that next-generation sequencing (PancreaSeq^®^) of pancreatic cystic fluid has a high sensitivity and specificity for differentiating between cystic lesions and advanced neoplasias or pNETS. The accuracy was increased by combining different markers, such as MAPK/GNAS and P53/SMAD4/CTNNB1/mTOR, reaching a sensitivity of 89% and specificity of 98% for advanced neoplasia. The inclusion of cytopathologic evaluation improved the sensitivity to 93% and maintained a high specificity of 95%. The study also pointed out the potential clinical applications of their results. Indeed, the addition of next-generation sequencing molecular techniques to international guidelines for IPMNs, such as American Gastroenterological Association [26] and IAP/Fukuoka [27] guidelines, increased the sensitivity of their analyses, which were 72% and 86%, respectively, to 96%, while specificities remained essentially the same. Another interesting result was the behavior of cystic lesions during follow-up depending on the mutations present. Serous cystic adenomas with TP53/TERT mutations exhibited interval growth, while pNETs with loss of heterozygosity of ≥3 genes tended to have distant metastasis. These results could guide the choice of follow-up modalities and personalize the global management of pancreatic cystic lesions. Finally, the fact that 98% of EUS-acquired samples were suitable for next-generation sequencing testing makes it an accessible technique.

### 2.5. The Role of Artificial Intelligence

Artificial intelligence (AI) is a generic term referring to computer-based learning techniques. Deep learning refers to neural networks into which EUS images and patterns are loaded with the purpose of developing methods for detecting patterns in anatomical or pathological features, differentiating pancreatic structures, or acting as a guide for EUS procedures by recognizing landmarks.

A recent review and meta-analysis conducted by Mohan et al. [28] evaluated the overall performance of neural network-based machine learning algorithms based on EUS image or video learning. Studies evaluating the accuracy of either detection of solitary pancreatic lesions or differentiation of chronic pancreatitis from malignancy were assessed and demonstrated a diagnostic accuracy of 85.5%, a sensitivity of 91.8%, a specificity of 84.6%, a positive predictive value of 87.4%, and a negative predictive value of 91.4%. Although premature for clinical application, this study demonstrates the high diagnostic performance of AI based on EUS image learning.

Early experimental clinical applications were tested by an AI system designed by the Wuhan EndoAngel Medical Technology Co. Ltd. (Wuhan, China) [29]. This deep-learning technology based on EUS images and video data in addition to real-time time-intensity curve analysis and CE-EUS, guided FNA for an adequate diagnostic tissue sampling by differentiating malignant, benign, and necrotic regions of the lesion.

Another deep-learning-based model, the BP MASTER^®^, was designed for real-time station recognition in pancreatic EUS. The study, conducted by Zhang et al. [30], reported positive results in which the accuracy of recognition was 94.2% and was comparable to EUS experts. As the station approach in pancreatic EUS evaluation is standardized [31] and the loss of the pancreatic frame is an important factor in misdiagnosis, this system could be used as an assistance tool for the operator as well as an important learning tool for trainees by providing systematic guidance. Indeed, the study results showed a significant improvement in station recognition in trainees by using this device.

In a systematic review, Goyal et al. [32] showed that different AI systems report variable accuracy rates for the evaluation of solid pancreatic lesions. The overall sensitivity of all AI systems in recognizing pancreatic malignancy ranged from 83 to 100%, with a specificity range of 50–99%, and an accuracy range of 80–97.5%. High values of specificity, sensitivity, and accuracy were also observed for the ability to differentiate between chronic pancreatitis and pancreatic cancer, including differentiating malignant from benign IPMNs. The authors pointed out that the AI-based system with the highest diagnostic odds ratio (OR) is the support vector machine system. This system is a human-supervised machine-learning process in which the machine uses EUS images to create data for classification and regression analysis. For training the learning model, EUS images are marked as belonging to one of two categories. A support vector machine training algorithm builds a model that assigns new examples to one category or the other. In addition to the diagnostic accuracy, the authors also see a possibility for screening for pancreatic malignancy in patients with chronic pancreatitis using the support vector machine system.

A more advanced approach for the application of AI was reported in a study from Ishikawa et al. [33] in which AI using contrastive learning was applied to compare the sensitivity, specificity, and accuracy of AI for assessing specimens acquired during fine needle biopsy (FNB) through macroscopic on-site evaluation (MOSE), to the assessment provided by an EUS expert. MOSE reflects the direct macroscopic evaluation of the core tissue obtained from EUS-FNB by the operator, which appears generally as a whitish solid material, in order to estimate the adequacy of the sample for further histological diagnosis during EUS-FNB. The results demonstrated sensitivity, specificity, and accuracy values of 90.34%, 53.5%, and 84.39%, respectively, and were comparable to those of MOSE performed by EUS experts.

The future perspective for AI will be to incorporate additional clinical data until AI becomes a “superhuman system” [2] capable of diagnosis and prognostic prediction in pancreatobiliary malignancy. Of course, more studies and data are necessary in order regulate the use of AI in this setting and to set clear guidelines about its application. An important limitation is the detection of rare types of pancreatic lesions, due to reduced AI training with EUS images as EUS images being the most frequently used input data. In addition, in this situation it is also a challenge to indicate the grounds on which clinical decisions were made. Some authors have called this the “black box problem” [2]. Details of the selected publications are shown in Table 1.

### 2.6. Screening in High-Risk Patients

Currently, PDAC screening is reserved for a so-called high-risk population, which is defined by the International Cancer of the Pancreas Screening Consortium as individuals with preneoplastic pancreatic cystic lesions, individuals with recent-onset diabetes, and patients with chronic pancreatitis, or when genetic syndromes or a family history of pancreatic neoplasms are present [34]. The role of genetic predisposition as a risk factor of PDAC was highlighted by Overbeek et al., who conducted a 13-year prospective study to investigate the yield of pancreatic cancer screening by EUS and MRI or MRCP in predisposed individuals vs individuals with no identified predisposing genetic mutation. Indeed, the diagnostic yield is non-existent in this latter population [35].

The current statement from the European Society of Gastrointestinal Endoscopy [36] is that EUS may be used in selected high-risk patients because of its accurate detection of small lesions, which could constitute early-stage pancreatic cancers. An older comparative prospective analysis from Harinck et al. [37] reported complementary roles for EUS imaging and magnetic resonance imaging (MRI). EUS is particularly sensitive for the early detection of small solid lesions, while MRI is very sensitive for the detection of small cystic lesions. In a recent study, Siegel et al. [38] reported a very high concordance between EUS and MRI with regard to worrisome features, but without significantly different clinical outcomes. This result confirmed earlier findings in another meta-analysis [39]. Regarding this, the International Cancer of the Pancreas Screening Consortium guidelines recommend screening with EUS and MRI, as well as a fasting blood glucose level or HbA1c at baseline, and then alternating annual screening with MRI or EUS and fasting blood sugar glucose or HbA1c. No consensus has been reached on whether and how to alternate EUS and MRI/magnetic resonance cholangiopancreatography (MRCP) [34]. There are no strong data to confirm when follow-up should be stopped or whether life-long surveillance is mandatory [12]. It is important to remember that EUS is an operator-dependent procedure, and that the sensitivity is not 100%. As the ideal screening modality is not yet fully agreed upon, research is moving towards biomarkers to detect microscopic precancerous lesions [23]. AI techniques could also to be considered in future perspectives in PDAC screening or surveillance, as suggested by Zhang et al. [30] in their study with the BP MASTER^®^.

## 3. Devices and Modalities for Tissue Acquisition

Although the current standard method for sampling of solid pancreatic lesions is still FNA, there are new data concerning FNB with MOSE. During FNA, a small amount of tissue is aspirated through a hollow needle in order to obtain material for cytological analysis, whereas FNB provides a larger amount of tissue that allows for assessment of the architecture and subsequent histological analysis. The needle tip design is different between FNA and FNB, and this is the reason why more tissue is available with FNB and why FNB can provide improved architectural preservation.

Rapid onsite cytopathological evaluation (ROSE) consists of the preparation of cytology slides, staining, and assessment of sample adequacy by a pathologist, onsite and directly in the procedure room. As cited in the previous paragraph, MOSE consists of direct macroscopic evaluation of the core tissue obtained from EUS-FNB by the operator.

### 3.1. MOSE

Mangiavillano et al. [40] studied the impact of MOSE in a recently published multicenter study that reported an overall diagnostic yield of 90% and, specifically among pancreatic lesions, a diagnostic accuracy for malignancy of 87.6%, sensitivity of 86.5%, specificity of 100%, positive predictive value of 100%, and negative predictive value of 40%. Therefore, this technique may represent a valid alternative when ROSE is not feasible. The analyses also showed that variables that were independently associated with diagnostic yield of MOSE included a larger needle diameter (20G vs. 25G, OR 11.64; 22G vs. 25G, OR 6.20) and three or more needle passes. These excellent pooled diagnostic accuracy rates in EUS-guided tissue acquisition by FNB using MOSE evaluation were also confirmed in a meta-analysis from Mohan et al. [41]. Interestingly, in the Mangiavillano study, there was no significant difference in diagnostic accuracy for MOSE associated with using different needle types (Procore vs. Aquire vs Echotip vs Sharkcore) [40].

These results were further fine-tuned by a randomized controlled noninferiority trial [42] in which no statistically significant differences were found between EUS-FNB with MOSE and conventional EUS-FNB in terms of diagnostic accuracy (90.0% vs. 87.8%). Nevertheless, the median number of passes was significantly lower in the EUS-FNB with MOSE group (1 vs. 3; *p* < 0.001). The authors concluded that MOSE reliably assesses sample adequacy and reduces the number of needle passes required to obtain the diagnosis with a 22 G FNB needle.

As previously mentioned, and as expected in the age of AI, comparative studies between MOSE evaluated by a human expert and AI have already been conducted [32].

MOSE is now being considered more often as an alternative to ROSE [43] because it saves the time and costs related to the presence of a pathologist in the room during endoscopy. Recently, Zhang et al. [44] conducted a prospective, randomized controlled trial in which they discussed the performance of self-ROSE, i.e., ROSE performed by a trained endoscopist after tissue sampling by FNA. The consistency between endoscopists and pathologists regarding sample adequacy and cytopathological diagnosis was good (*p* < 0.001 and *p* < 0.05, respectively), and diagnostic accuracy and sensitivity were both significantly increased during EUS-FNA in the self-ROSE group compared to the group without ROSE evaluation at all.

### 3.2. Needle Types for Tissue Acquisition

A multicenter randomized controlled trial reported by Crinò et al. [45] confirmed the noninferiority of EUS-FNB without ROSE compared to FNB with ROSE in solid pancreatic lesions when new-generation FNB needles are used. In their conclusion, the authors did not recommend ROSE in routine clinical practice. In addition, significantly higher tissue core rates were obtained by EUS-FNB without ROSE, with a significantly shorter mean sampling procedural time.

Finally, a recent review and network meta-analysis conducted by Gkolfakis et al. [46] compared the diagnostic accuracy of different FNB needles. Data were assessed from 16 randomized controlled trials and showed that Franseen and Fork-tip needles (new-generation FNB needles) significantly outperformed reverse-bevel needles. Regarding size, the best-performing devices in sensitivity analysis were 22-gauge. It is important to note that, in contrast to what was reported by Crinò et al. [45], the authors showed that no needle type was significantly superior when ROSE was available. On the other hand, in a meta-analysis from Li et al. [47], the authors reported no difference in diagnostic accuracy or tissue cores rates between FNB needles or FNA needles for solid pancreatic lesions.

In an international prospective trial, Al-Haddad et al. [48] looked at the question of whether using a flexible needle for cystic pancreatic lesions could have an impact on diagnostic accuracy, patient management, or volume of fluid aspiration for tumor markers. There were no statistically significant differences between 19 G, 19 G flex, or 22 G needles. However, there seems to be a trend toward more success in FNA sampling of cystic pancreatic lesions in the pancreatic head or uncinate process by using a flexible needle.

### 3.3. Sampling Techniques and Number of Needle Passes

Sampling techniques were assessed in the single-center randomized trial from Mendoza Ladd et al. [49]. Three sampling techniques, including slow pull, dry suction, and wet suction, were compared in terms of cellularity score, blood contamination, and number of passes needed for diagnosis, and no significant differences were observed. Three passes were enough to obtain a histological diagnosis in most patients. However, these results were not confirmed by Chen et al. [50], who showed that, compared with the dry suction technique, wet suction yielded a significantly higher diagnostic accuracy, better specimen adequacy score and cellularity score, and lower blood contamination score. Tong et al. [51] reported similar results. Specifically, a modified wet suction technique resulted in significantly better quality of specimen, histological, and first-pass diagnostic yields and comparable safety compared with the dry suction technique. Due to the fact that both studies, Mendoza Ladd et al. [49] and Tong et al. [51], involved only a small number of included patients, it is not possible at this time to recommend one technique over the other.

Jin et al. [52] focused on the ideal number of needle passes with the different tissue acquisition techniques. By using a 22G ProCore™ needle (Cook Medical, National Technology Park, Limerick, Ireland), they recommend at least three passes using the standard suction technique and at least four passes when using the stylet slow-pull technique to yield a diagnostic accuracy of >90%. Mendoza Ladd et al. [49] reported a diagnostic accuracy, after two and three passes, of 82% and 100%, respectively, with the Sharkcore™ needle (Medtronic, Minneapolis, MN) and 67% and 91%, respectively, with the Acquire™ needle (Boston Scientific, Natick, MA, USA).

**Figure 2:**
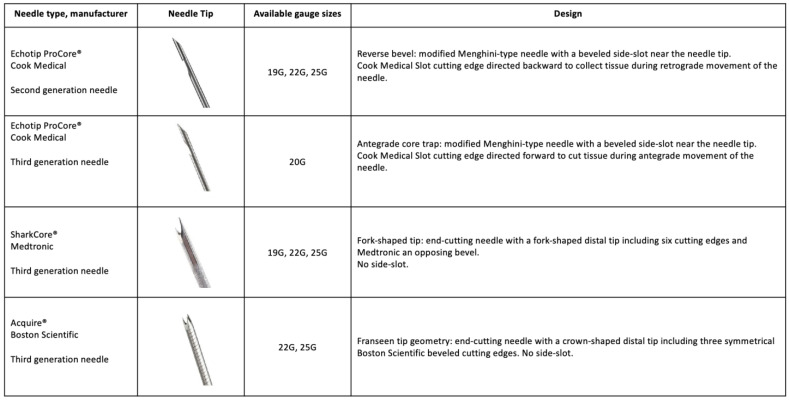
Design and key features of available EUS-FNB needles (adapted from Kovacevic et al. [54] and Polkowski et al. [55]).

In the last update of the European Society of Gastrointestinal Endoscopy guidelines in 2021 concerning the sampling of pancreatic solid masses, FNA and FNB needles are equally recommended [53]. However, newer generation FNB needles (i.e., forward facing bevels, fork tip, or crown tip) are recommended when the intended aim is to obtain core tissue and when ROSE is not available [53]. For better diagnostic accuracy with FNA, the guidelines also provide information about the technical aspects on how to process the obtained material. They suggest dividing the material into smears and liquid-based cytology or processing the whole EUS-FNA material as liquid-based cytology, depending on local experience. Table 2 summarizes the reviewed literature, while Figure 2 demonstrates the characteristics of different available FNB needles.

## 4. Techniques for Local EUS-Guided Treatment

### 4.1. Radiofrequency Ablation

Radiofrequency ablation (RFA) is a recent technique that has gradually gained a significant place in the management of pancreatic cystic neoplasms and pNETs. Barthet et al. [56] conducted a prospective, multicenter, non-randomized study that demonstrated that RFA is a promising alternative for the management of pNETs ranging from 1 to 2 cm and in cystic pancreatic lesions with worrisome features or high-risk stigmata. The study was conducted with a prospective follow-up of 3 to 5 years. In their experience, 85.7% of the pNETs had completely disappeared after EUS-RFA treatment. Regarding cystic pancreatic lesions, only 40% disappeared completely, in contrast to 100% of the mural nodules, which were classically considered as high-risk stigmata. One limitation of RFA is the uncertainty concerning the management of RFA treatment failure as well as long-term efficacy. The authors suggested long-term surveillance by EUS paired with CE-EUS, as it was shown to be useful in solid pancreatic lesions.

Garg et al. [57] reported results from a meta-analysis that compared EUS-RFA with another ablative therapy: ethanol ablation. For pNETs, they reported comparable outcomes in terms of effectiveness and safety. Clinical success rates after EUS-RFA and EUS with ethanol ablation were 85.2% and 82.2%, respectively. Adverse effects were also comparable for both techniques (14.3% with EUS-RFA and 11.7% with EUS with ethanol ablation).

According to the results of a systematic review, Gollapudi et al. [58] have suggested that EUS-RFA could also play a role as a complementary therapeutic approach in unresectable pancreatic cancers or for downstaging in borderline resectable patients. In another systematic review, Spadaccini et al. [59] pointed out the limited risk of serious adverse effects with this technique.

### 4.2. Additional Therapeutic Procedures

EUS-guided techniques have also found application in additional therapeutic procedures for PDAC, such as fiducial insertion for stereotactic body radiation therapy. A recent meta-analysis by Patel et al. [60] evaluated technical aspects of EUS-guided fiducial placement specifically for PDAC. Recently, preloaded needles have become available to make this procedure easier [4]. This study reported an overall technical success rate of 96.2%, a migration rate of 4.3%, and an adverse event rate of 4.8%. The authors drew attention to the fact that this technique may be performed during the same session with other EUS -guided procedures, such as tissue sampling or coeliac plexus block, to achieve pain control. However, there is no evidence of an improvement in mortality rates compared to traditional fiducial placements. A more marginal option could also be Hydrogel™ (TraceIt Fiducial Marker; Augmenix Inc., Waltham, MA, USA), an injectable solution, placed locally close to the pancreatic lesion, that works as a liquid fiducial [61]. Another EUS-assisted procedure is EUS-guided fine-needle tattooing, performed to help to precisely localize the pancreatic lesion, in case of surgical resection [61].

## 5. Conclusions

EUS is an indispensable tool in the management of pancreatic lesions at all levels. Diagnostic potential is enhanced by modalities such as CE-EUS and elastography; and these techniques have also been shown to aid in the selection of the best window for tissue acquisition. Additionally, major advancements have taken place regarding needle design, further increasing diagnostic accuracy. The addition of AI is also proving to be a valuable tool and could potentially become part of future routine practice. EUS-guided techniques can facilitate local treatment for preneoplastic lesions and complement therapeutic management for PDAC.

## Figures and Tables

**Table 1:** Summary of the studies describing the recent developments in Artificial Intelligence systems.

Study (Year)	Study Type	Objective	Data type	AI	Results	Application	Limitations
Mohan et al., 2022	Meta-analysis 11 studies 2001–2020	Overall performance of AI in: -Diagnosis and characterization of solid PL -Differentiate PC from non-neoplastic tissue -Differentiate malignancy from CP -Diagnosis of PC	-EUS elastography -EUS images -EUS—videos -CEH-EUS	Fractal-based quantitative analysis NN algorithm SVM	-Overall accuracy 85.8% -Sens 91.8% -Spec 84.6% -PPV 87.4% -NPV 91.4% -Heterogeneity 57%	Superior diagnostic results with the combination of AI and newer core-biopsy needles in EUS evaluation of solid masses	-Heterogeneity -Absence of prospective data
Yu et al., 2022	Case report	Guiding punction of pancreatic masses by differentiating cancerous, non-cancerous, and necrotic regions	N/A	Deep CNN		Improving the diagnostic accuracy of EUS FNA	
Zhang et al., 2020	Cross-over 8 participants	BP-MASTER^®^ -Test the performance of classifying the previously learned stations of pancreatic EUS -Pancreatic tissue and blood vessel segmentation	-EUS images -EUS videos	Deep learning	-Classification accuracy 86% -Comparable accuracy between endoscopists and AI -Improvement of trainee’s accuracy for classification and segmentation	Shortening the pancreatic EUS learning curve Improving EUS quality control	-Duodenal bulb station non studied
Goyal et al., 2022	Systematic review 11 studies	Study the effectiveness of AI with EUS in the diagnosis of pancreatic cancer -Differentiating PC from CP -Differentiating malignant from benign IPMNs	-Retrospective EUS images and videos -Real time collected EUS images	ANN CNN SVM	Performance in recognition of pancreatic malignancy: -Sens 83–100% -Spec 50–99% -Accuracy 80–97.5% -PC vs. CP *ANN* -Sens 88–100% -Spec 50–94% -SVM -Sens 96% -Spec 93% -Accuracy 94% *CNN* -Sens 90% -Spec 75% Benign vs. malignant IPMNs *CNN* -Sens 95.7% -Spec 92.6% -Accuracy 94%	-Improvement of pancreatic malignancy recognition even in presence of chronic pancreatitis -SVM method simpler and highly performant	
Ishikawa et al., 2022	Retrospective	-Study the usefulness of AI in predicting the EUS-FNB sample quality for histopathological examination	-Stereomicroscopic images of EUS-FNB specimens	CNN and deep learning Contrastive learning	-AI evaluation using contrastive learning is comparable to MOSE performed by EUS experts -Diagnostic accuracy with deep learning not as high as MOSE performed by experts	Increasing the objectivity of the evaluation	Small sample size

AI artificial intelligence, ANN artificial neuronal networks, CEH-EUS contrast enhanced EUS, CNN convolutional neural networks, CP chronic pancreatitis, EUS endoscopic ultrasound, FNA fine needle aspiration, FNB fine needle biopsy, IPMN intraductal papillary mucinous neoplasm, MOSE macroscopic on-site evaluation, NN neural network, NPV negative predictive value, PC pancreatic cancer, PL pancreatic lesions, PPV positive predictive value, Sens sensitivity, Spec specificity, SVM Support vector machine.

**Table 2:** Summary or recent studies on different types of EUS needles.

Study (Year)	Study Type	Study Sample	Objective	Localization	Device	Results
Mangiavillano et al., 2021	Multicenter study	378 patients	Diagnostic yield and accuracy of MOSE with different needle sizes	Pancreatic and extra-pancreatic lesions	Procore^®^ Acquire^®^ Echotip ultra^®^ Sharkcore^®^	Association with the diagnostic yield of MOSE -larger needle diameter -≥3 needle passes
Gkolfakis et al., 2022	Network meta-analysis	16 RCTs 1934 patients	Compare the diagnostic accuracy of available FNB needles for sampling of solid pancreatic lesions	Solid pancreatic lesions	22/25G FNA 20 G Side-fenestrated forward-facing bevel 22 G Franseen 19/22/25 G Fork-tip 21/22 G Menghini-tip 22/25 G Reverse bevel	Franseen 22 G -AUC 0.89 for accuracy 0.94 for adequacy Fork-tip needles 22 G -0.76 for accuracy -0.73 for adequacy 25 G Franseen and 25 G Fork-tip needles were not superior to 22 G reverse-bevel needles
Li et al., 2022	Meta-analysis	18 RCTs 2718 patients	Compare the diagnostic value and safety of FNA and FNB—needles	Pancreatic and extra-pancreatic lesions		-Solid pancreatic lesions: no difference in diagnostic accuracy -Overall gastrointestinal lesions: better diagnostic accuracy with FNB needles
Al-Haddad et al., 2021	Multicenter prospective randomized trial	250 patients	Impact of three FNA needles on -diagnostic accuracy -accrue fluid for tumor markers	Pancreatic cystic lesions	19 G Fle x19 G 22 G	-Overall success rate for aspiration: higher for 19 G Flex and 22 G compared with 19 G -No difference in the percentage of cyst volume aspirated by needle type

AUC area under the curve, EUS endoscopic ultrasound, FNA fine needle aspiration, FNB fine needle biopsy, MOSE Macroscopic one-site evaluation, RCT randomized controlled trial.

## Abbreviations

AI	artificial intelligence
CEA	carcinoembryonic antigen
CE-EUS	contrast enhanced EUS
CEH-EUS	contrast enhanced harmonic mode EUS
EUS	endoscopic ultrasound
EUS-FNB	endoscopic ultrasound-guided fine needle biopsy
FNA	fine needle aspiration
FNB	fine needle biopsy
IPMN	intraductal papillary mucinous neoplasm
MOSE	macroscopic on-site evaluation
MRCP	magnetic resonance cholangiopancreatography
MRI	magnetic resonance imaging
nCLE	needle-based confocal laser endomicroscopy
OR	Odds ratio
PDAC	pancreatic ductal adenocarcinoma
pNETs	pancreatic neuroendocrine tumors
RFA	radiofrequency ablation
ROSE	rapid on-site evaluation
